# Leptin and TGF-β1 Downregulate PREP1 Expression in Human Adipose-Derived Mesenchymal Stem Cells and Mature Adipocytes

**DOI:** 10.3389/fcell.2021.700481

**Published:** 2021-07-13

**Authors:** Andreina Bruno, Caterina Di Sano, Hans-Uwe Simon, Pascal Chanez, Angelo Maria Patti, Serena Di Vincenzo, Paola Dino, Vittoria D’Esposito, Pietro Formisano, Francesco Beguinot, Elisabetta Pace

**Affiliations:** ^1^Institute for Biomedical Research and Innovation (IRIB), National Research Council, Palermo, Italy; ^2^Institute of Pharmacology, University of Bern, Bern, Switzerland; ^3^Institute of Biochemistry, Medical School Brandenburg, Neuruppin, Germany; ^4^Department of Clinical Immunology and Allergology, Sechenov University, Moscow, Russia; ^5^Laboratory of Molecular Immunology, Institute of Fundamental Medicine and Biology, Kazan Federal University, Kazan, Russia; ^6^Department of Respiratory Diseases CIC Nord INSERM, INRAE, C2VN, Aix Marseille University, Marseille, France; ^7^Department of Health Promotion Sciences Maternal and Infantile Care, Internal Medicine and Medical Specialties (PROMISE), University of Palermo, Palermo, Italy; ^8^URT Genomics of Diabetes, Institute of Experimental Endocrinology and Oncology, National Research Council, Naples, Italy; ^9^Department of Translational Medicine, Federico II University of Naples, Naples, Italy

**Keywords:** adipose tissue, adipocytes, adipocyte-derived stem cells, leptin, PREP1, TLR4, immune system, TGF-beta1

## Abstract

Adipose tissue is widely recognized as an extremely active endocrine organ producing adipokines as leptin that bridge metabolism and the immune system. Pre-B-cell leukemia homeobox (Pbx)-regulating protein-1 (PREP1) is a ubiquitous homeodomain transcription factor involved in the adipogenic differentiation and insulin-sensitivity processes. Leptin, as pleiotropic adipokine, and TGF-β, known to be expressed by primary pre-adipocytes [adipose-derived stem cells (ASCs)] and mature differentiated adipocytes, modulate inflammatory responses. We aimed to assess for the first time if leptin and TGF-β interfere with PREP1 expression in both ASCs and mature differentiated adipocytes. Human ASCs were isolated from subcutaneous adipose liposuction and, after expansion, fully differentiated to mature adipocytes. In both ASCs and adipocytes, leptin and TGF-β1 significantly decreased the expression of PREP1, alone and following concurrent Toll-like receptor 4 (TLR4) activation. Moreover, in adipocytes, but not in ASCs, leptin increased TLR4 and IL-33 expression, whereas TGF-β1 enhanced TLR4 and IL-6 expression. Taken together, we provide evidence for a direct regulation of PREP1 by leptin and TGF-β1 in ASCs and mature adipocytes. The effects of leptin and TGF-β1 on immune receptors and cytokines, however, are limited to mature adipocytes, suggesting that modulating immune responses depends on the differentiation of ASCs. Further studies are needed to fully understand the regulation of PREP1 expression and its potential for the development of new therapeutic approaches in obesity-related diseases.

## Introduction

Until 25 years ago, white adipose tissue (WAT) was considered an energetic storage tissue only. Many years of experimental and clinical research have provided evidence for the crucial role of WAT in regulating, besides metabolism, the immune system (Cinti, 2019; Cox et al., 2019). WAT does not only produce cytokine-like hormones (adipokines), it also hosts a wide array of resident and infiltrating immune cells, namely, T and B lymphocytes, macrophages, neutrophils, and eosinophils, that maintain tissue homeostasis in lean subjects (Huh et al., 2014). Subcutaneous (peripheral) and visceral (central) WATs have been proven to serve as an accessible and abundant source of adipose-derived stem cells (ASCs), which provides the possibility to apply tissue engineering techniques. For instance, ASCs, as pluripotent stem cells, can be differentiated not only into multi-lineage mesodermal cell types (adipocytes, myocytes, chondrocytes, and osteoblasts) but also into cells of endodermal and ectodermal origins (Bunnell et al., 2008; Tobita et al., 2011; Cawthorn et al., 2012). Taking into account these characteristics, ASCs represent a good *in vitro* model to investigate physiological and pathological mechanisms in WAT. Adipokines, with inflammatory and anti-inflammatory activities, are known to modulate the immune system. Particularly, IL-6, IL-33, and leptin participate in the maintenance of the metabolic balance by increasing the insulin sensitivity and preserving adipose tissue homeostasis (Rana et al., 2019; Xu et al., 2019). Leptin is one of the most relevant adipokines that regulates the crosstalk between adipocytes and the immune system (Bruno et al., 2005b; Conus et al., 2005; Francisco et al., 2018). Leptin receptors are widely expressed in cells of the innate and adaptive immune systems, regulating the inflammatory response in several diseases (Pérez-Pérez et al., 2017).

Interestingly, there is evidence that leptin can induce the perpetuation of inflammation by maintaining immune cell survival (Bruno et al., 2005b; Conus et al., 2005); on the other hand, leptin can protect bronchial and nasal epithelial cells (Bruno et al., 2005a, 2009, 2014, 2019), maintaining and/or restoring epithelial homeostasis. IL-6 and IL-33 are cytokines induced by Toll-like receptor 4 (TLR4) activation that play a key role in the development of immune inflammatory responses in several organs, such as the lung (Hochdörfer et al., 2011; Martínez-González et al., 2013; Zhao et al., 2021). Moreover, both IL-6 and IL-33 are involved in anti-inflammatory immune process and in several physiological systems, such as skeletal muscle for IL-6: release of IL-6 increased from skeletal muscle after prolonged exercise is known to be associated with stimulation of hypertrophic muscle growth and myogenesis of muscle stem cells in human ASCs (Serrano et al., 2008; Muñoz-Cánoves et al., 2013; Seo et al., 2018). On the other hand, IL-33 maintains type 2 innate lymphoid cells (ILC2) and reduces the risk of metabolic syndrome (Brestoff et al., 2015). IL-33 is able to counter excessive inflammation in adipose tissue responses by targeting immune cells expressing the receptor suppression of tumorigenicity 2 receptor (ST2) for IL-33. Furthermore, mice lacking ST2 or IL-33 develop increased adiposity and worsened metabolic profiles, as well as IL-33 treatment triggers the expansion of a group of Fox3 + ST2 + Tregs and attenuates adipose tissue inflammation (Bantulà et al., 2021). Moreover, the metabolic activity of the epicardial adipose tissue is associated with elevated IL-33 (Gruzdeva et al., 2018).

Leptin and IL-33 are both involved in basophil degranulation (Koketsu et al., 2013), whereas IL-33 upregulates leptin receptor at both the mRNA and surface protein levels in human basophils, indicating that leptin may be a key molecule mediating the effects of adipocytes on inflammatory cells, such as basophils, by binding to leptin receptor and activating the cellular functions (Suzukawa et al., 2011).

It is well known that adipose tissue is an endocrine organ, but also an immune organ, as it is physiologically infiltrated by innate immune cells. The health and homeostasis of adipose tissue is important to contribute for maintaining a regular and physiological immune response. Adipose tissue exhibits important roles in regulating energy metabolism and insulin sensitivity (Huang and Xu, 2021). Nevertheless, when obesity is present, the low-grade state of inflammation in both omental and subcutaneous adipose tissues often results in impaired glucose metabolism and may contribute to the development of insulin resistance and weight gain. Moreover, obesity and insulin resistance further compromise the already impaired immune systems (Andrade et al., 2021).

Both leptin and insulin are of equal importance in the pathophysiology of obesity and type 2 diabetes (T2DM; Rehman et al., 2018). On the other hand, leptin and insulin can also increase the secretion of pro-inflammatory cytokines by immune cells (Zakharova et al., 2019; Frasca et al., 2020).

Pre-B-cell leukemia homeobox (Pbx)-regulating protein-1 (PREP1) plays a role in metabolic disorders. It is a homeodomain transcription factor from the three-amino acid loop extension (TALE) superclass of proteins that dimerizes with Pbx1, which is implicated in modulating embryonic development (Berthelsen et al., 1998) and metabolism (Cimmino et al., 2019). Several studies performed in mice report that PREP1 is involved in glucose and lipid metabolism regulation of the muscle, liver, and fat cells, suggesting that PREP1 may provide an early contribution to obesity and T2DM pathogenesis (Ciccarelli et al., 2016; Oriente et al., 2018). Moreover, PREP1 deficiency induces protection from diabetes (Oriente et al., 2008), reduces inflammatory responses, and increases insulin sensitivity in adipose tissue (Liotti et al., 2018). The relatively high efficiency of ASCs in pluripotency can be explained, at least partially, by their high expression of TGF-β (Sugii et al., 2010), which is known to regulate differentiation and remodeling. Furthermore, a recent study investigating the expression of angiogenic markers during the osteogenesis of ASCs reported an increased expression of regulatory genes, such as JUN, FOS, and MAPK3, involving TGF-β pathways (Shaik et al., 2019). Moreover, TGF-β takes part in obesity-associated inflammation and in T2DM mechanism (Frühbeck et al., 2020; Zhang et al., 2020), as well as in cancer together with PREP1 (Risolino et al., 2014). Cytokine production from ILC2 is controlled also by TGF-β that inhibits adipogenesis and maintains insulin sensitivity in adipocytes (Lumeng et al., 2007; Tsurutani et al., 2011).

However, the precise mechanisms whereby adipocyte and various immune cells crosstalk with each other to aggravate obesity-induced adipose inflammation and metabolic dysregulation remain poorly defined. The present study was performed to establish the role of leptin and TGF-β in the regulation of PREP1 expression in both primary human ASCs and mature differentiated adipocytes. The expression of additional molecules involved in the interaction with the immune system was also investigated.

## Materials and Methods

### Reagents

Collagenase A (from *Clostridium histolyticum* cat no. 11088793001) was from Roche (Indianapolis, IN, United States). Recombinant human insulin (cat no. 26360.01) and dexamethasone (cat no. 18660.01) were from Serva (Heidelberg, Germany).^1^ Indomethacin (cat no. 70270) was from Cayman (Ann Arbor, MI, United States). Recombinant human leptin (398-LP) and recombinant human TGF-β1 (240-B) were purchased from R&D System (Minneapolis, MN, United States). Monoclonal PREP1 (B-2): sc-25282 antibody, rabbit polyclonal TLR4 (H-80): sc-10741 antibody, HBD2 (FL-64): sc-20798 antibody, and goat polyclonal anti-leptin receptor (Ob-R M-18): sc-1834 antibody were from Santa Cruz Biotechnology, Inc. (Dallas, TX, United States). Monoclonal CD284 (TLR4) PE (cat no 12-9917) antibody was from eBioscience (San Diego, CA, United States). Monoclonal anti-CD34, anti-CD45, anti-CD73, anti-CD90, and anti-CD105 antibodies, pure or FITC/PE conjugate, were purchased from MACS, Miltenyi Biotec (Germany). Normal donkey (NDS, 017-000-121), normal goat serum (NGS, 005-000-121), and TRITC-conjugated donkey anti-rabbit IgG (711-025-152) were from Jackson Immunoresearch (West Grove, PA, United States). Alexa Fluor^®^ 488-conjugated goat anti-mouse IgG (A 11029) was from Thermo Fisher Scientific (Waltham, MA, United States). TRIzol reagent was from Life Technologies (Carlsbad, CA, United States), iScript cDNA Synthesis kit was from BioRad (Hercules, CA, United States), and FAM-labeled probe and primers were from Applied Biosystems (Foster City, CA, United States).

### Liposuction Procedure, Isolation of ASCs From Adipose Tissue, and Cell Culture

Adipose tissue was collected by liposuction aspiration of subcutaneous fat from non-obese and non-diabetic anonymous subjects [n = 7; 5 females; mean age, 39; mean body mass index (BMI), 29]. All subjects were non-smokers and without hypertension, cardiovascular or metabolic disease, cancer, or chronic pulmonary diseases. They signed their informed consensus, and the Ethics Committee of Policlinico, Giaccone Hospital, Palermo, Italy (authorization reference number 12/2014) in agreement with the Helsinki Declaration approved the study. Every adipose sample was kept at room temperature (RT) for less than 24 h prior to use, and ASCs were isolated as previously described (Bunnell et al., 2008). Briefly, approximately 500 mL liposuction aspiration was washed extensively in a sterile bottle to remove contaminating heterogeneous cell population, namely, circulating blood cells, fibroblasts, pericytes, and endothelial cells, by two subsequent washing (1 h, RT) in a ratio of 1:1 with phosphate-buffered saline (PBS) containing 5% penicillin/streptomycin and 10% fungizone (P/S/F). The stromal vascular fraction was then separated from the lower layer of heterogeneous cells and discharged. The sample was shaken for 1 h at 37°C in PBS containing 2% P/S, 10% F, and 0.075% collagenase type I and subsequently neutralized by adding in the ratio 1:1, 5 mL of D-MEM with 20% heat-inactivated fetal bovine serum (FBS). After centrifugation at 2,000 rpm for 5 min, the pellet was washed in lysis buffer and incubated for 10 min on ice and then washed and incubated in two wells of a 24-well plate at 37°C with 5% CO_2_ in complete proliferation medium: D-MEM cell growth medium high glucose (4.5 g/L D-glucose), 3.7 g/L NaHCO_3_, W/O sodium pyruvate, L-glutamine, and phenol red (ECM0106L; Euroclone, Milan, Italy) with 1% antibiotics mixture (penicillin, streptomycin sulfate, and amphotericin B, 03-033-1; Biological Industries Israel Beit Haemek Ltd., Kibbutz Beit-Haemek, Israel), 1% L-glutamine 200 mM, 1% sodium pyruvate, and 20% FBS. The yield of ASCs (undifferentiated pre-adipocytes) obtained from liposuction sample after digestion was patient-dependent. Usually, we obtained approximately 2 × 10^5^ cells in a single well of a 12-well plate or approximately 1 × 10^5^ cells in a single well of a 24-well plate for an amount of approximately 500 or 250 mg of liposuction aspiration fluid adipose tissue, respectively. Seventy-two hours after plating, D-MEM cell growth medium 20% FBS was replaced with fresh D-MEM cell growth medium 10% FBS. This latter was changed every 3 days. At this point, there were two options: ASCs were allowed to reach between 80 and 90% confluence and induced to differentiate; otherwise, ASCs were directly stimulated as briefly described: ASCs were cultured in D-MEM cell growth medium 10% FBS in the presence and absence of recombinant human leptin (0.001–2.5 μM) and recombinant human TGF-β1 (40–1.600 pM) for 24 h. The best active concentrations were 0.5 μM for leptin and 400 pM for TGF-β1. For the TLR4 pathway activation, we used endotoxin, LPS (purchased from Sigma-Aldrich, St. Louis, MO, United States; 10 μg/mL) for 24 h. Cells and supernatants (stored at –80°C after centrifugation until ELISA test) were then collected for further analyses.

### Adipogenic Differentiation

Adipose-derived stem cells display multipotent characteristics, meaning that despite they derive from an adult human tissue, they maintain the ability to differentiate into multilineage cell types (adipogenic, osteogenic, chondrogenic, and neuronal differentiation) (Bunnell et al., 2008), leading to their interest for their potential therapeutic value in the regenerative medicine field. We differentiated ASCs only in mature adipocytes. Specific differentiation in mature adipocytes was directly induced by adding the D-MEM differentiation medium: it means that D-MEM cell growth medium 20% FBS was enriched with 1 μM dexamethasone, 1 μM insulin, and 50 μM indomethacin. When 1 × 10^5^ ASCs per well seeded in a 24-well plate were at 80–90% confluence, we started to induce the adipogenic differentiation by adding 1 mL complete D-MEM differentiation medium. The medium was carefully changed twice per week. In 28 days, the cell differentiation process was completed. At this day, mature adipocytes either were tested for red oil staining (below described) or were cultured in complete D-MEM differentiation medium, in the presence and absence of recombinant human leptin and recombinant human TGF-β1 at the same conditions used for ASCs. Cells and supernatants (stored at –80°C after centrifugation until ELISA test) were then collected for further analyses.

### Red Oil Staining

The accumulation of neutral lipids was detected by staining the cells on a slide as described: cells, adherent in a plasticware plate, were fixed with 10% paraformaldehyde for 90 min at 37°C; afterward, cells were stained in a solution of Oil Red O solution (Oil Red O solution—0.5% in isopropanol (O1391) from Sigma-Aldrich, St. Louis, MO, United States) for 30 min at 37°C. The percentage of cells undergoing adipogenesis was calculated by image analyzer microscope (Axioskop-2-Zeiss, Jena, Germany).

### Flow Cytometer

The multipotent ASCs cell differentiation process from mature differentiated adipocytes was also assessed by specific flow cytometric surface antigen expression analyses of both ASCs and mature differentiated adipocytes. The mesenchymal stromal cells express CD73, CD90, and CD105 and lack the expression (≤2% positive) of CD45, CD34, and other surface antigens (Dominici et al., 2006). Furthermore, ASCs and adipocytes were stimulated as described above; cells were then fixed with 4% paraformaldehyde, permeabilized with PBS containing 1% FBS, 0.3% saponin, and 0.1% Na azide, and stained with anti-human PREP1, TLR4, HBD2, and leptin receptors primary antibodies and the appropriate PE- and FITC-conjugated secondary antibodies. Marker expression was evaluated using a FACSCalibur flow cytometer (Becton Dickinson, Mountain View, CA, United States). Non-immune IgGs at the same concentration as the primary antibody were used as negative controls. Percentages of positive cells were determined from forward and sideways scatter patterns. Non-specific binding and background fluorescence were quantified by analyzing the negative controls.

### Immunofluorescence

To maintain the integrity of marker membrane, ASCs were seeded with proliferation medium on glass coverslips, previously sterilized with ethanol 70%, and allowed to attach for 72 h before stimulation or before adding differentiation medium. After stimulation of both ASCs and adipocytes, cells were fixed with 4% paraformaldehyde for 15 min, permeabilized with 0.1% Triton X-100 in PBS for 5 min, and blocked in 0.1% Tween 20 in PBS (TPBS) containing 2% BSA and 3% normal goat serum for PREP1, CD34, CD73, and CD90 or 3% normal donkey serum for TLR4, for 1 h, before staining. Cells were then incubated with anti-PREP1, anti-CD34, anti-CD73, anti-CD90, and anti-TLR4 (all, 1:50) antibodies in TPBS containing 2% BSA and 1.5% normal goat or 1.5% normal donkey serum, respectively, for 2 h at RT. Incubation with the appropriate secondary antibodies Alexa Fluor 488 or TRITC-conjugated (1:200) in TPBS containing 2% BSA and 1.5% normal goat or 1.5% normal donkey serum, respectively, was performed for 30 min. Glass coverslips were mounted on slides in Vectashield (Vector Laboratories, Burlingame, CA, United States) with 406-diamidine-2-phenylindole-dihydrochloride (DAPI) to visualize the nuclei. Staining of the samples was observed and evaluated with an Axioskop-2-Zeiss microscope/software. Control slides were prepared using an irrelevant antibody of the same isotype and at the same concentration as the specific primary, and no significant staining or fluorescence signals were detected.

### Quantitative Real-Time Reverse Transcription-Polymerase Chain Reaction

The whole RNA was isolated from ASCs and from mature adipocytes using TRIzol reagent (Life Technologies) following the manufacturer’s instruction. Then, 1 μg of RNA was reverse-transcribed to cDNA, using iScript cDNA Synthesis kit (BioRad). Quantitative real-time PCR by TaqMan Assay to evaluate the gene expression of PREP1, leptin, leptin receptors, TGF-β1, TLR4, IL-6, and IL-33 was performed. Transcripts were carried out with StepOnePlus Real-time PCR System (Applied Biosystems, Foster City, CA, United States) using specific FAM-labeled probe and primers (pre-validated TaqMan Gene expression assay for PREP1, Hs00231814_m1, for leptin, Hs00174877-m1, for leptin receptors, Hs00174492-m1, for TGF-β1, Hs00998133-m1, for TLR4, Hs00152939-m1, for IL-6, Hs00985639-m1, and for IL-33, Hs01125943-m1; Applied Biosystems). Gene expression was normalized to glyceraldehyde-3-phosphate dehydrogenase (GAPDH) (pre-validated TaqMan Gene expression assay for GAPDH, Hs03929097g1; Applied Biosystems) in each case (Haslett et al., 2002). The results were obtained with the comparative Ct method (2^–ΔΔ*Ct*^), and untreated cells were used as reference samples.

### Cytokines Release by ASCs and Mature Differentiated Adipocytes

Supernatants were recovered, centrifuged, and stored at –80°C until assayed. IL-6, IL-8, IL-33, IL-1β, and TNF-α were measured with a commercially available ELISA Kit (Duo Set ELISA; R&D Systems, Minneapolis, MN, United States) following the manufacturer’s instructions.

### Statistical Analysis

The analysis of results was performed from 3 to 7 independent biological replicates. The numbers of independent experiments are indicated in the figure legends. Analysis of variance (ANOVA) was used for testing differences between means. The possible association between categorical variables was evaluated by the accurate Fisher’s exact test. A *p*-value < 0.05 was considered statistically significant.

## Results

### Characterization of ASCs and Differentiated Adipocytes

**FIGURE 1:**
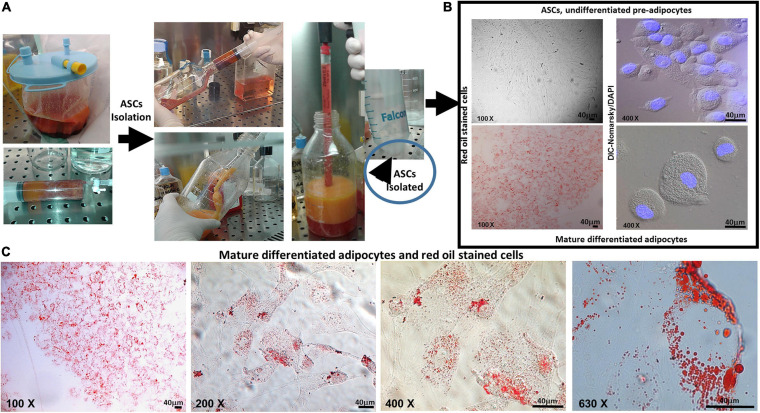
**(A)** Isolation from liposuction sample. **(B)** Upper and lower left, ASCs and mature differentiated adipocytes, both stained with red oil (×100): upper left figure shows ASCs stained, negative for red oil test, and adherent in plasticware plate; lower left shows mature differentiated adipocytes stained, positive for red oil test, and adherent in plasticware plate. **(B)** Upper and lower right, differential interference contrast (DIC-Nomarski) combined with DAPI (×400) for ASCs and mature differentiated adipocytes is shown. **(C)** Mature differentiated adipocytes with red oil staining (magnification at ×100, ×200, ×400, and ×630, scale bar = 40 μm) are shown. Axioskop-2-Zeiss microscope.

**FIGURE 2:**
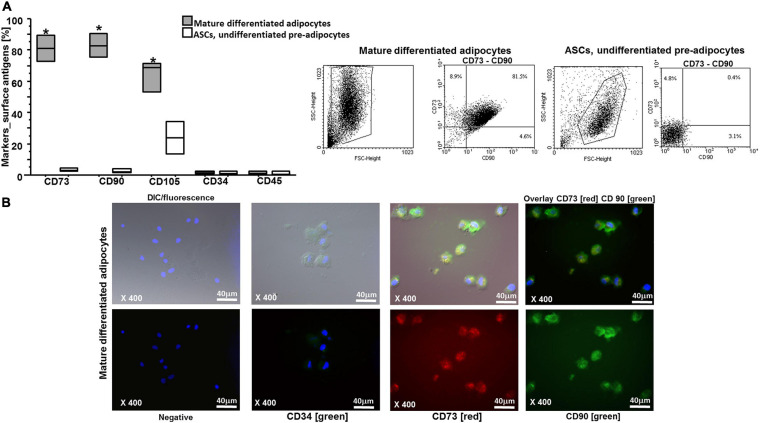
**(A)** Flow cytometry analysis on ASCs and on mature differentiated adipocytes for antigen surface markers: the results are shown as box-plots with medians (lines inside the boxes), mature differentiated adipocytes significantly expressed CD105, CD73, and CD90 in comparison with ASCs (*p = 0.0272 for CD105, **p < 0.0001 for CD73, and CD90; *n* = 3 experiments). Analysis of variance (ANOVA), Fisher’s PLSD. Right, representative examples of flow cytometric analysis in ASCs and in adipocytes. The numbers indicate the percentage of double positive cells for CD73 and CD90. **(B)** Representative immunofluorescence for antigen surface markers on mature differentiated adipocytes is shown: higher, differential interference contrast (DIC-Nomarski) combined with DAPI and fluorescence; lower, DAPI with fluorescence; negative, CD34 (green), CD73 (red), and CD90 (green) (magnification at ×400, scale bar = 40 μm). Axioskop-2-Zeiss microscope.

After the isolation from the liposuction sample (different devices), the undifferentiated multipotent ASCs were cultured and expanded by using specific proliferation culture medium (Figure 1A). ASCs were adherent to the plasticware surface (Figure 1B, upper left), as one of the three minimal criteria required by the *Mesenchymal and Tissue Stem Cell Committee of the International Society for Cellular Therapy* (Dominici et al., 2006). The other two criteria are the specific surface antigen (Ag) expression and the multipotent differentiation potential. The undifferentiated multipotent ASCs were expanded, and parts of them were fully differentiated into mature adipocytes (Figure 1B, lower left). We assessed the full differentiation in adipocytes by red oil staining and specific surface markers. By red oil staining, we observed the characteristic red neutral lipid droplets in fully mature differentiated adipocytes (Figures 1B, lower left, C). In contrast, the undifferentiated pre-adipocytes were red oil negative (Figure 1B, upper left). By flow cytometry and immunofluorescence, we observed an increased expression of CD73, CD90, and CD105 in differentiated adipocytes compared with ASCs (*p* < 0.0001, *p* < 0.0001, *p* = 0.0243, respectively), whereas the expression of CD34 and CD45 markers was not significantly different between the two cytotypes and less than 2% (Figure 2). Figure 2B shows the DIC-Nomarski overlay with fluorescence to appreciate the morphology of both the pre-adipocytes and adipocytes.

### Leptin and TGF-β1 Decrease PREP1 Levels but Increase TLR4 Surface Expression

**FIGURE 3:**
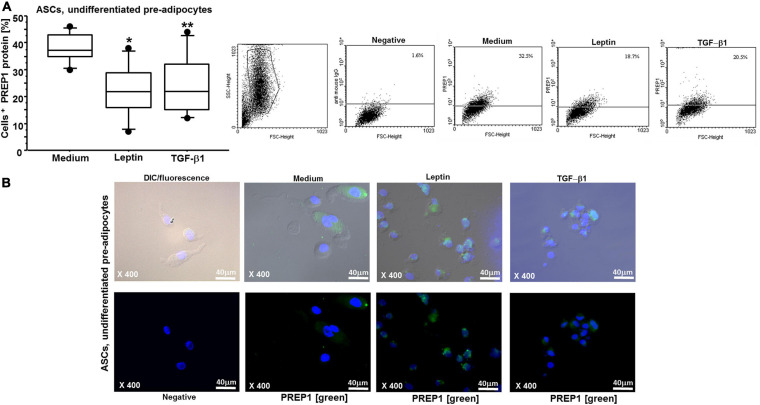
**(A)** Flow cytometry analysis on ASCs for PREP1: both leptin (0.05 μM) and TGF-β1 (400 nM) significantly reduced PREP1 expression (*p = 0.0149 and **p = 0.0318, respectively); the results are shown as box-plots with medians (lines inside the boxes), 25th and 75th percentiles (limits of boxes), and the 10th and 90th percentiles (whiskers) (*n* = 7 experiments). Analysis of variance (ANOVA), Fisher’s PLSD. Right, representative examples of flow cytometric analysis. The numbers indicate the percentage of positive cells for PREP1. **(B)** Representative immunofluorescence for PREP1 (green) on ASCs is shown: higher, differential interference contrast (DIC-Nomarski) combined with DAPI and fluorescence; lower, DAPI with fluorescence (magnification at ×400, scale bar = 40 μm). Axioskop-2-Zeiss microscope.

**FIGURE 4:**
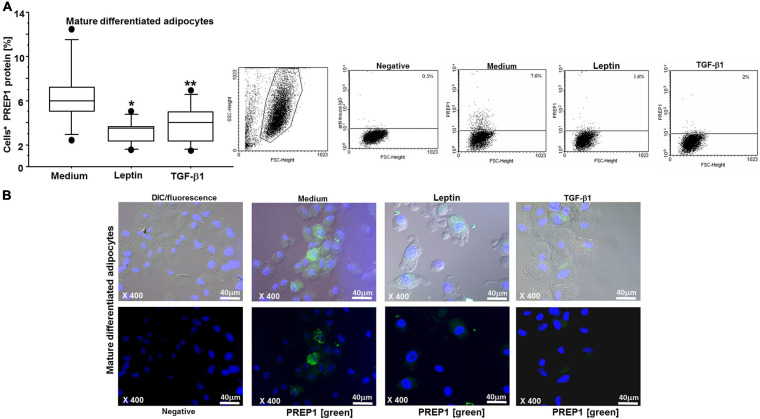
**(A)** Flow cytometry analysis on mature differentiated adipocytes for PREP1: both leptin (0.05 μM) and TGF-β1 (400 nM) significantly reduced PREP1 expression (*p = 0.0138 and **p = 0.0461, respectively); the results are shown as box-plots with medians (lines inside the boxes), 25th and 75th percentiles (limits of boxes), and the 10th and 90th percentiles (whiskers) (*n* = 6 experiments). Analysis of variance (ANOVA), Fisher’s PLSD. Right, representative examples of flow cytometric analysis. The numbers indicate the percentage of positive cells for PREP1. **(B)** Representative immunofluorescence for PREP1 (green) on mature differentiated adipocytes is shown: higher, differential interference contrast (DIC-Nomarski) combined with DAPI and fluorescence; lower, DAPI with fluorescence (magnification at ×400, scale bar = 40 μm). Axioskop-2-Zeiss microscope.

**FIGURE 5:**
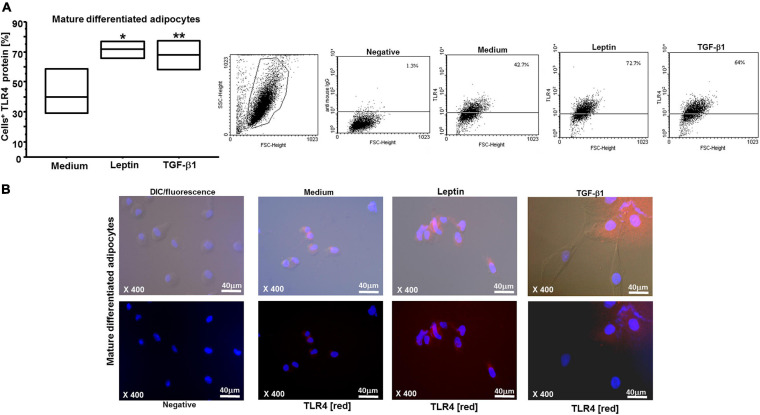
**(A)** Flow cytometry analysis on mature differentiated adipocytes for TLR4: both leptin (0.05 μM) and TGF-β1 (400 nM) significantly increased TLR4 expression (*p = 0.0178 and **p = 0.0325, respectively); the results are shown as box-plots with medians (lines inside the boxes) (*n* = 4 experiments). Analysis of variance (ANOVA), Fisher’s PLSD. Right, representative examples of flow cytometric analysis. The numbers indicate the percentage of positive cells for TLR4. **(B)** Representative immunofluorescence for TLR4 (red) on mature differentiated adipocytes is shown: higher, differential interference contrast (DIC-Nomarski) combined with DAPI and fluorescence; lower, DAPI with fluorescence (magnification at ×400, scale bar = 40 μm). Axioskop-2-Zeiss microscope.

**FIGURE 6:**
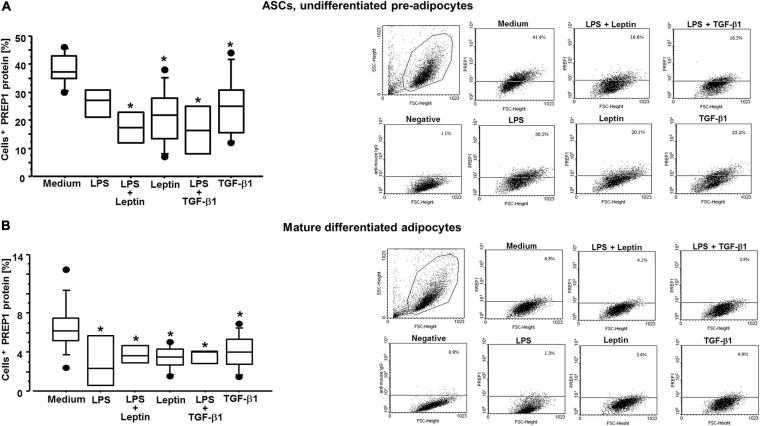
**(A)** Flow cytometry analysis on ASCs for PREP1 with and without LPS for TLR4 activation: both leptin (0.05 μM) and TGF-β1 (400 nM) significantly reduced PREP1 expression with or without LPS (10 μg/mL); medium vs. leptin + LPS (*p* = 0.0135); medium vs. leptin (*p* = 0.0034); medium vs. TGF-β1 + LPS (*p* = 0.0100); medium vs. TGF-β1 (*p* = 0.0168); the results are shown as box-plots with medians (lines inside the boxes), 25th and 75th percentiles (limits of boxes), and the 10th and 90th percentiles (whiskers), at least *n* = 3 experiments. Analysis of variance (ANOVA), Fisher’s PLSD. Right, representative examples of flow cytometric analysis. The numbers indicate the percentage of positive cells for PREP1. **(B)** Flow cytometry analysis on mature differentiated adipocytes for PREP1 with and without LPS for TLR4 activation: both leptin (0.05 μM) and TGF-β1 (400 nM) significantly reduced PREP1 expression with or without LPS (10 μg/mL); medium vs. LPS (*p* = 0.0136); medium vs. leptin + LPS (*p* = 0.0484); medium vs. leptin (*p* = 0.0032); medium vs. TGF-β1 + LPS (*p* = 0.0319); medium vs. TGF-β1 (*p* = 0.0116); the results are shown as box-plots with medians (lines inside the boxes), 25th and 75th percentiles (limits of boxes), and the 10th and 90th percentiles (whiskers), at least *n* = 3 experiments. Analysis of variance (ANOVA), Fisher’s PLSD. Right, representative examples of flow cytometric analysis. The numbers indicate the percentage of positive cells for PREP1.

By flow cytometry and immunofluorescence, we observed that both leptin and TGF-β1 significantly reduced PREP1 expression (^∗^*p* = 0.0149 and ^∗∗^*p* = 0.0318, respectively) in both ASCs (Figure 3) and mature differentiated adipocytes (^∗^*p* = 0.0138 and ^∗∗^*p* = 0.0461, respectively) (Figure 4). Furthermore, as TLR4 has been implicated in adipose tissue inflammation, the effects of both leptin and TGF-β1 on TLR4 expression were evaluated. Both leptin and TGF-β1 significantly increased TLR4 expression (^∗^*p* = 0.0178 and ^∗∗^*p* = 0.0325, respectively) in mature differentiated adipocytes (Figure 5), but not in ASCs. To test the possible role of TLR4 in PREP1 regulation, the effect of LPS, the main agonist of TLR4, also in combination with leptin or TGF-β1, on the expression of PREP1 in both ASCs and adipocytes was assessed by flow cytometry. In ASCs, LPS only when combined with leptin or TGF-β1 versus medium significantly reduced PREP1 expression (*p* = 0.0135 and *p* = 0.0100, respectively) (Figure 6A). In adipocytes, both LPS alone versus medium and combined with leptin or TGF-β1 significantly reduced PREP1 expression (*p* = 0.0136, *p* = 0.0484, and *p* = 0.0319, respectively) (Figure 6B).

### Leptin and TGF-β1 Act Independently in ASCs and Mature Adipocytes

**FIGURE 7:**
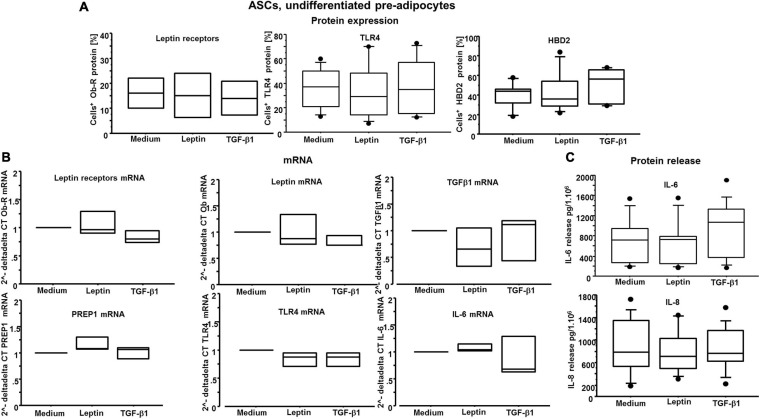
**(A)** Flow cytometry analysis on ASCs for leptin receptors, TLR4 and HBD2: there are no significant differences among the conditions. **(B)** *n* = 3 experiments; quantitative real-time PCR of leptin and its receptors, TGF-β1, PREP1, TLR4, and IL-6 transcripts: there are no significant differences among the conditions. **(C)** At least n = 7 experiments; IL-6 and IL-8 release by ASCs: there are no significant differences among the conditions.

**FIGURE 8:**
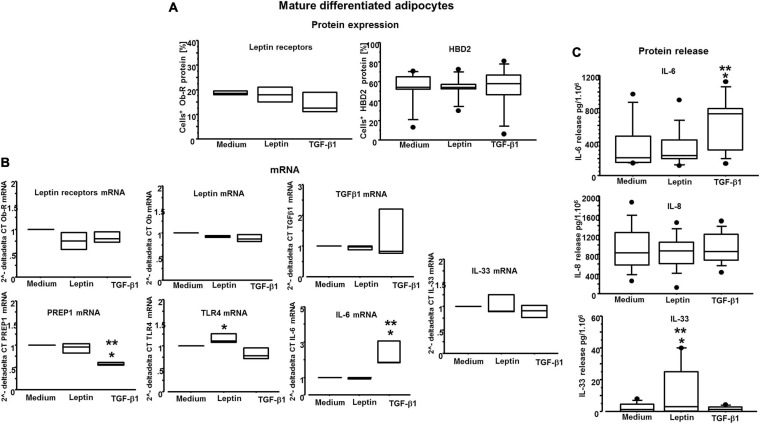
**(A)** Flow cytometry analysis on mature differentiated adipocytes for leptin receptors and HBD2: there are no significant differences among the conditions. **(B)** *n* = 3 experiments; quantitative real-time PCR of leptin and its receptors, TGF-β1 and IL-33 transcripts: there are no significant differences among the conditions; RT-PCR of PREP1 transcripts is significantly reduced only by TGF-β1 (400 nM) versus medium (*p = 0.0008) and versus leptin (0.05 μM) (**p = 0.0022); RT-PCR of TLR4 transcripts is significantly increased only by leptin (0.05 μM) versus TGF-β1 (400 nM) (*p = 0.0143); RT-PCR of IL-6 transcripts: it is significantly increased only by TGF-β1 (400 nM) versus medium (*p = 0.0203) and versus leptin (0.05 μM) (**p = 0.0179). **(C)** At least n = 7 experiments; IL-6, IL-8, and IL-33 release by mature differentiated adipocytes: for IL-8, there are no significant differences among the conditions, whereas IL-6 was significantly increased only by TGF-β1 (400 nM) versus medium (*p = 0.0486) and versus leptin (0.05 μM) (**p = 0.0199), and IL-33 was significantly increased only by leptin (0.05 μM) versus medium (*p = 0.0486) and versus TGF-β1 (400 nM) (*p = 0.0424). Analysis of variance (ANOVA), Fisher’s PLSD.

Previous studies have shown that the expression of leptin and TGF-β1 is inversely correlated in airway remodeling processes in asthma and allergic rhinitis (Bruno et al., 2009, 2014). It has been previously demonstrated that TGF-β1 reduces leptin receptor expression in bronchial epithelial cells, and that leptin reduces TGF-β1 release *in vitro* (Christensen et al., 2018). Here, we investigated whether both leptin and TGF-β1 affected leptin receptor/leptin expression, and whether, conversely, leptin affected TGF-β1 mRNA expression in ASCs and mature adipocytes. We did not observe similar findings as previously described in bronchial epithelial cells (Figures 7A,B, 8A,B), supporting the concept that leptin and TGF-β1 act independently in ASCs and adipocytes.

### IL-6 Gene Expression and Protein Release Are Increased by TGF-β1 but Not by Leptin

As we wanted to gain insight on the role of leptin and TGF-β1 in modulating inflammatory responses by both ASCs and adipocytes, the effects of both leptin and TGF-β1 were explored assessing the releases of antimicrobial peptide beta-defensin 2 (HBD2) protein and multiple cytokines, namely, TNF-α, IL-1β, IL-6, IL-8, and IL-33. HBD2 protein expression was not significantly affected by either leptin or TGF-β1, on both ASCs and adipocytes (Figures 7A, 8A). In mature differentiated adipocytes, IL-6 gene expression was significantly increased by TGF-β1 versus medium and leptin (*p* = 0.0203 and *p* = 0.0179, respectively) (Figure 8B), whereas PREP1 gene expression was significantly increased by TGF-β1 versus medium and leptin (*p* = 0.0008 and *p* = 0.0022, respectively) (Figure 8B). Moreover, in mature differentiated adipocytes, IL-6 protein release was significantly increased by TGF-β1 only but not leptin (Figure 8C). On the other hand, IL-33 protein release was significantly increased by leptin and not TGF-β1 (Figure 8C), but IL-33 mRNA expression was not significantly different. In ASCs, both IL-33 mRNA and protein were not detectable. Moreover, TNF-α and IL-1β were neither detectable in ASCs nor in adipocytes. Finally, the expression of IL-8 was not significantly different following leptin or TGF-β1 stimulation (Figures 7C, 8C).

## Discussion

Although pluripotent ASCs clearly hold immense therapeutic promise and are therefore at the forefront of regenerative medicine, the developmental relevance of these cells remains poorly established (Cawthorn et al., 2012). Moreover, ASCs represent a good *in vitro* model to investigate the mechanisms responsible for obesity in humans. Obesity is an epidemic disease characterized by a chronic low-grade state of inflammation; obesity and T2DM are both genetically defined and of absolute concern globally, in almost all countries in the world (Christensen et al., 2018; Patti et al., 2018; Assaad Khalil et al., 2019; Boachie et al., 2020). In this study, we investigated the modulation of PREP1 expression in ASCs and mature differentiated adipocytes by the cytokines leptin and TGF-β1. We report the following new findings: (1) leptin and TGF-β1 significantly decrease PREP1 protein expression in both ASCs and mature adipocytes. (2) TGF-β1 significantly reduces PREP1 mRNA expression in adipocytes. (3) Leptin and TGF-β1 increase TLR4 expression in mature adipocytes but not in ASCs. (4) TLR4 activation by LPS decreases PREP1 protein expression in adipocytes. (5) In mature adipocytes, TGF-β1 increases both IL-6 mRNA and protein release, whereas leptin increases IL-33 release.

Alterations of differentiation, proliferation, and endocrine function, such as those that occur in conditions of excess (obesity), deficit (lipodystrophy), and/or altered regional distribution of adipose tissue, are frequently associated with insulin resistance frameworks predisposed to T2DM (Capel et al., 2018). Leptin and insulin are equally important in the metabolic features of the pathophysiology of obesity (Rehman et al., 2018), and PREP1 plays a primary role in organogenesis and metabolism (Cimmino et al., 2019). PREP1 has been identified as gene encoding for a homeodomain transcription factor, which plays a relevant activity in glucose and lipid homeostasis (Oriente et al., 2008). In this regard, it has been observed that hypomorphic heterozygous mice (PREP1i/+), expressing low levels of PREP1 protein, are protected from streptozotocin-induced diabetes, exhibit improved insulin sensitivity, and reduce visceral adipose tissues (Oriente et al., 2013). Increased PREP1 levels lead to reduced adipocyte differentiation (Liotti et al., 2018).

We demonstrate in this study that leptin, TGF-β1, or TLR4 activation, by decreasing PREP1 levels, can limit the adipogenesis and, at the same time, may maintain adipose tissue homeostasis. This latter concept is also important in understanding the different basal PREP1 expression levels in ASCs compared with mature differentiated adipocytes. PREP1 is a notable transcription factor involved in embryogenesis and first organ development. Its downregulation in zebrafish and its deletion in mice were associated with embryonic lethality (Fernandez-Diaz et al., 2010). Based on these concepts, it is conceivable that PREP1 expression is higher (basal PREP1 expression, mean = 38%) in ASCs than in mature differentiated adipocytes (basal PREP1 expression, mean = 6%). In this context, a previous study suggested that IL-33 is produced by M2 macrophages, which sustain adipose tissue homeostasis.

Interestingly, IL-33 contributes to maintain the lean state through the activation of anti-inflammatory M2 macrophages, and in humans, low serum IL-33 levels are associated with high BMI (Liew et al., 2016; Bantulà et al., 2021).

In contrast, classically activated M1 macrophages produce IL-1β, TNF-α, and IL-8 (Rana et al., 2019). Furthermore, a recent study performed in diabetic mice^*ob/ob*^ demonstrated increased expression of TNF receptor genes, whereas IL-33, cholecystokinin, plasminogen activator, IL-1β, and serine peptidase inhibitor genes were downregulated (Yıldız et al., 2020). In agreement with this study, we observed that IL-1β and TNF-α are not expressed in normal ASCs and mature differentiated adipocytes. IL-8 is equally expressed in both ASCs and adipocytes, whereas leptin alone is able to significantly increase IL-33 protein release by mature differentiated adipocytes.

We used ASCs derived from non-obese and non-diabetic patients, both males and females, with a range of BMI in overweight. In clinical practice, recombinant leptin is an important experimental and therapeutic tool: recent studies on metreleptin (recombinant methionyl human leptin) have suggested its efficacy in patients with T2DM (Vasandani et al., 2017) as leptin acts directly on liver cells to reduce the production of glucose and to increase the sensitivity of insulin (Rehman et al., 2018).

To date, innovative hypoglycemic therapies have not only metabolic but also pleiotropic effects, directing the diabetologist no longer to the evaluation of glycemia but above all in the “tailored” management of the patient and the complications of the metabolic syndrome (Patti et al., 2020). The metabolic obese patient, thanks to the new therapeutic approach that targets also the immune system and the cellular senescence as causal factors in obesity-related inflammation, has a new hope for treating obesity-related metabolic dysfunction and its complications (Palmer et al., 2019). On the other side, TLR4 pathway dysregulation may also account for obesity-associated inflammation and insulin resistance (Zhang et al., 2020). In this regard, both beneficial and detrimental effects of TLR4 pathway activation have been demonstrated on the functionality of adipose tissue in experimental mouse studies (Tao et al., 2017). During a chronic high-saturated fat diets challenge, TLR4 supports adipose inflammation that in turn protects insulin sensitivity through promoting healthy adipose tissue remodeling and expansion. On the other hand, during an acute challenge with saturated fatty acids, a negative role of TLR4 as a mediator of insulin resistance in the adipocytes was observed.

Here, although the direct impact of leptin, TGF-β1, or TLR4 activation (by adding LPS) in the differentiation processes of ASCs was not assessed, we speculate that leptin, TGF-β1, or TLR4 activation reduced PREP1, a molecule with a known fundamental role in the adipocyte differentiation process, can also reduce adipogenesis. The impaired capacity of omental compared with subcutaneous adipose tissues for remodeling and expanding through hyperplasia is thought to be associated with visceral adiposity with insulin resistance and metabolic health. In this context, several experimental studies have been performed to test this hypothesis. In omental adipose tissue, local factors including inflammatory cytokines and members of the TGF-β superfamily inhibited adipogenesis and also hormonal factors, such as glucocorticoids regulate adipogenesis (Lee and Fried, 2014; Almuraikhy et al., 2016). Furthermore, TGF-β receptor mRNA levels were more robustly induced by low concentrations of dexamethasone, and ASCs produced higher levels of TGF-β ligands, which act in an autocrine/paracrine loop to inhibit adipogenic differentiation (Lee et al., 2019). Our results on PREP1 decreased by TGF-β1 are in line with these previously published observations and allow us to speculate that the health of WAT could be maintained by leptin and TGF-β1. Leptin and TGF-β1 are thereby protective cytokines in the context of physiological adipogenesis and healthy adipose tissue, as they increase TLR4 and decrease PREP1 expression. Our finding that TLR4 activation in mature adipocytes reduced PREP1 expression further supports the assumption that activation of the TLR4 pathway has beneficial metabolic effects. The data obtained in ASCs, showing that TLR4 activation further reduces the expression of PREP1 in cells co-stimulated with leptin or TGF-β1, are in support of this concept.

Leptin does not seem to induce TLR4 pathway activation as we observed no increased HBD2 expression or increased IL-8, IL-1β, TNF-α, and IL-6 expression levels. The lack of increased HBD2 protein expression is consistent with previous observations reporting that leptin, not alone, but together with other cytokine, enhances IL-1β-induced HBD2 secretion and mRNA expression in keratinocytes *via* the NF-kB pathway in epidermal keratinocytes (Kanda and Watanabe, 2008). In contrast to leptin, TGF-β1 can increase IL-6 mRNA expression and IL-6 protein release, as recently reported in the field of obesity and metabolic syndrome (Warbrick and Rabkin, 2019). IL-6 is also considered as a myokine, produced by the skeletal muscle during physical activity that counteracts the inflammatory adipokines produced by the adipose tissue of the sedentary subjects (Pedersen and Febbraio, 2012). Exercise and adipose tissue are associated with elevated plasma concentrations of IL-6 (Ferrandi et al., 2018), which counteracts obesity, insulin resistance, and T2DM. The increase in systemic concentrations of IL-6 is associated with the adipose tissue being the main source of this cytokine (Herder et al., 2005). IL-6 exhibits systemic effects on the liver, adipose tissue, and immune system. IL-6 also mediates the crosstalk between intestinal L cells and pancreatic islets: high concentrations of IL-6 in response to exercise stimulate glucagon-like peptide-1 (GLP-1) secretion from intestinal L cells and from pancreatic alpha cells, improving insulin secretion and blood sugar (Pedersen and Febbraio, 2012). In metabolic models, the beneficial effects of IL-6 were maintained, and the neutralization of IL-6 resulted in an additional increase in blood sugar and a decrease in GLP-1 in the pancreas. IL-6 also prevents the induction of insulin resistance and the deterioration of glucose homeostasis (Lazar, 2005). IL-6 can adapt the metabolism to physical exercise and obesity during pro-inflammatory states. This interaction between insulin sensitive tissues and insulin-producing cells is mediated by IL-6, which acts on L and alpha cells to promote the secretion and production of GLP-1.

## Conclusion and Take-Home Message

The role of leptin and TGF-β1 in reducing PREP1 expression, with and without activation of TLR4, might be interpreted as a protective role of a healthy adipose tissue, to prevent (primary prevention) the switch to an unhealthy adipose tissue characterized by pathological adipogenesis, insulin resistance, and infiltrating M1 macrophages producing the pro-inflammatory adipokines TNF-α and IL-1β. Leptin, TGF-β1, and TLR4 pathway through PREP1 reduction or increased IL-6 expression could be promising new biological markers and therapeutic targets in obesity-related diseases.

## Dedication

This work is dedicated to the memory of Antonella Siena.

## Data Availability Statement

The original contributions presented in the study are included in the article/supplementary material, further inquiries can be directed to the corresponding author/s.

## Ethics Statement

The studies involving human participants were reviewed and approved by Ethic Committee of Policlinico-Giaccone Hospital-Palermo Italy (authorization reference number 12/2014) in agreement with the Helsinki Declaration approved the study. The patients/participants provided their written informed consent to participate in this study.

## Author Contributions

AB: conception and design of the study, acquisition, interpretation, and analysis of data, and writing the manuscript. CD, SD, PD, VD’E, and PF: analysis and interpretation of the data. H-US and EP: analysis and interpretation of the data and revising critically the manuscript for important intellectual content for final approval of the version to be submitted. PC and FB: providing approval for publication of the content. AP: interpretation of the data and writing the manuscript. All authors contributed to the article and approved the submitted version.

## Conflict of Interest

The authors declare that the research was conducted in the absence of any commercial or financial relationships that could be construed as a potential conflict of interest.

## Footnotes

1 www.serva.de
